# Inhaled Pollutants: The Molecular Scene behind Respiratory and Systemic Diseases Associated with Ultrafine Particulate Matter

**DOI:** 10.3390/ijms18020243

**Published:** 2017-01-24

**Authors:** Hussein Traboulsi, Necola Guerrina, Matthew Iu, Dusica Maysinger, Parisa Ariya, Carolyn J. Baglole

**Affiliations:** 1 Department of Medicine, McGill University, Montreal, QC H4A 3J1, Canada; Hussein.Traboulsi@mail.mcgill.ca (H.T.); matthew.iu@mail.mcgill.ca (M.I.); 2 Department of Pathology, McGill University, Montreal, QC H4A 3J1, Canada; Necola.guerrina@mail.mcgill.ca; 3 Department of Pharmacology & Therapeutics, McGill University, Montreal, QC H3G 1Y6, Canada; Dusica.maysinger@mcgill.ca; 4 Department of Chemistry, McGill University, Montreal, QC H3A 2K6, Canada; parisa.ariya@mcgill.ca

**Keywords:** air pollution, epigenetics, chronic obstructive pulmonary disease, particulate matter, aryl hydrocarbon receptor, nuclear factor-κB

## Abstract

Air pollution of anthropogenic origin is largely from the combustion of biomass (e.g., wood), fossil fuels (e.g., cars and trucks), incinerators, landfills, agricultural activities and tobacco smoke. Air pollution is a complex mixture that varies in space and time, and contains hundreds of compounds including volatile organic compounds (e.g., benzene), metals, sulphur and nitrogen oxides, ozone and particulate matter (PM). PM_0.1_ (ultrafine particles (UFP)), those particles with a diameter less than 100 nm (includes nanoparticles (NP)) are considered especially dangerous to human health and may contribute significantly to the development of numerous respiratory and cardiovascular diseases such as chronic obstructive pulmonary disease (COPD) and atherosclerosis. Some of the pathogenic mechanisms through which PM_0.1_ may contribute to chronic disease is their ability to induce inflammation, oxidative stress and cell death by molecular mechanisms that include transcription factors such as nuclear factor κB (NF-κB) and nuclear factor (erythroid-derived 2)-like 2 (Nrf2). Epigenetic mechanisms including non-coding RNA (ncRNA) may also contribute towards the development of chronic disease associated with exposure to PM_0.1_. This paper highlights emerging molecular concepts associated with inhalational exposure to PM_0.1_ and their ability to contribute to chronic respiratory and systemic disease.

## 1. Introduction

Air pollutants are released into the atmosphere and can cause significant harm to humans. According to the World Health Organization (WHO), around 3.5 million people die each year because of urban and indoor air pollution, making this a leading environmental health risk. Air pollution is now recognized as a human carcinogen and is regarded as major risk factor for acute and chronic diseases including cardiovascular disease (CVD), liver fibrosis and chronic respiratory diseases such as asthma and chronic obstructive pulmonary disease (COPD). More than 25% of premature deaths associated with air pollution are respiratory in nature [1].

The sources of air pollution are numerous and include anthropogenic sources such as transportation (e.g., vehicle exhaust), factory emissions (e.g., industries, coal-fired power plants), combustion (e.g., biomass, cigarette smoke) and agriculture (e.g., fertilizer and animal waste). Air pollution can also come from natural sources such as volcanoes, forest fires and dust storms. Regardless of the origin, air pollution is a toxic mixture of gases and particulate matter (PM) composed of organic chemicals (polycyclic aromatic hydrocarbons (PAH)), metals (iron, nickel), gases (ozone), biological agents (plant pollen, endotoxins, bacteria) and minerals (quartz, asbestos) [2,3]. The main gaseous contaminants are carbon monoxide and dioxide (CO and CO_2_ respectively), nitrogen dioxide (NO_2_), ozone (O_3_) and sulfur dioxide (SO_2_). Other important gaseous pollutants are ammonia and volatile organic compounds (VOCs; e.g., methane, benzene and chlorofluorocarbon). It is the inhalation of PM that is often associated with increased morbidity and mortality, the collective incidence and severity of which are strongly influenced by the duration and amount of PM exposure [4]. According to the WHO, it is estimated that exposure to PM causes about 16% of lung cancer deaths, 11% of COPD deaths, and more than 20% of ischaemic heart disease and stroke. PM is a complex airborne mixture of solid, liquid or mixed-phase particles composed of organic, inorganic and organometallic compounds suspended in air, and its composition varies in space and time. In terms of aerodynamic diameter, PM can be classified according to their size: PM_10_: coarse particles less than 10 µm in diameter; PM_2.5_: fine particles less than 2.5 µm; and PM_0.1_: ultrafine particles (UFPs) smaller than 100 nm, which includes the nanoparticles (NPs). Of these, PM_0.1_ have the potential to exert significant harm, as particles of this size can escape broncho-mucociliary action and scavenging by alveolar macrophages. These particles also penetrate deep into the respiratory tract (i.e., the alveolus) where they can be absorbed by the blood stream. Deposition patterns have revealed that the lungs are the primary target, although particles could be detected in other organs like the liver, kidney, heart and brain [5]. Studies in healthy human volunteers using Technegas, an ultrafine dispersion of Technetium-labelled carbon (99 mTc) particles approximately 4–20 nm in diameter, revealed that the PM_0.1_ remained in the lung up to 6 h after installation although some were detected in the blood immediately after inhalation [6]. Furthermore, these particles have a very large effective surface area where chemical interactions can occur and may be important biologically, as PM_0.1_ cross cell membranes and directly interact with cellular structures. Indeed, animal and in vitro studies have shown that acute exposures to PM_0.1_ may cause changes in lung function and airway inflammation, and could enhance allergic responses, increase vascular thrombogenic effects, alter endothelial function, affect heart rate variability and accelerate atherosclerosis [7].

**Figure 1:**
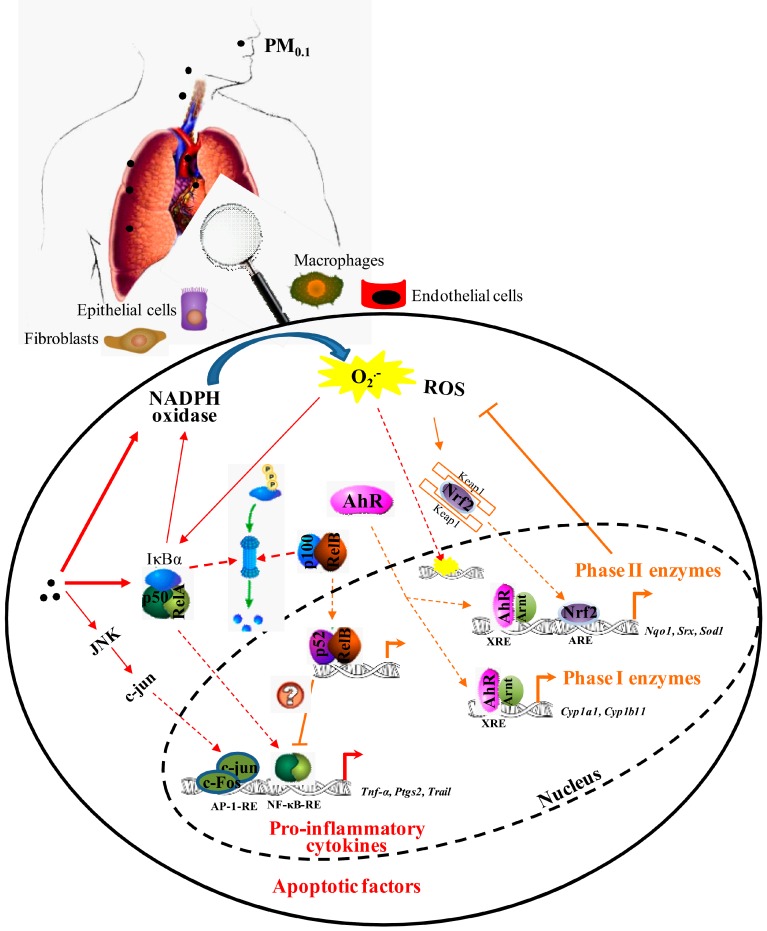
The molecular mechanisms associated with inflammatory and oxidative stress in response to chronic exposure to PM_0.1._ PM, particulate matter; ROS, generation of reactive oxygen; JNK, c-Jun kinase; AhR, Aryl hydrocarbon receptor; Nrf2, nuclear factor (erythroid-derived 2)-like 2; ARE, antioxidant response element; XRE, xenobiotic response element; AP-1-RE, AP-1 response element; NF-κB-RE, NF-κB response element; RelA, v-rel avian reticuloendotheliosis viral oncogene homolog A or p65; RelB, reticuloendotheliosis viral oncogene homologue B; NADPH, nicotinamide adenine dinucleotide phosphate-oxidase; Arnt, aryl hydrocarbon receptor nuclear translocator. Solid line: activation; dashed line: translocation; red line: inflammatory/oxidant pathway; red dashed line: anti-inflammatory/ anti-oxidant pathway.

During the last decade, it has become increasingly clear that the health impacts of PM_0.1_ are significantly affected by physical and chemical processes such as size, gas-particle partitioning, hygroscopicity, liquid-liquid phase separation, redox kinetics, surface tension, viscosity, molecular configuration, active sites, surface properties, and chemical composition. Although there is increasing assumption that PM_0.1_ are deleterious to human health, very little is known about the cellular, molecular and genetic/epigenetic alterations that may affect the incidence/severity of chronic disease in individuals exposed to PM_0.1_. In this review, we highlight emerging evidence of key biological and signal transduction pathways that trigger inflammation and oxidative stress in response to PM_0.1_ exposure as important contributors to the development of chronic respiratory and CVD (Figure 1).

## 2. Pathogenic Processes Implicated Following Inhalation of PM_0.1_

### 2.1. Inflammation

Acute inflammation is a self-limiting response to tissue injury [8] or can be part of the host response to invading pathogens [9]. Inflammation is induced by stress (chemical, physical), bacteria and viruses or environmental pollutants (e.g., cigarette smoke, air pollution) [10,11]. However, the persistence of initiating factors and/or inadequate resolution of the inflammatory response can itself lead to tissue damage and thus drives many pathogenic processes including carcinogenesis and autoimmunity [9]. The ability of PM to induce an inflammatory response is dependent on a number of factors, including the size, although particles of all sizes are capable of inducing an inflammatory response [12]. However, the dependence on size alone for overall toxicity—including inflammation—is varied. Some in vitro studies have indicated that smaller particles (those near the PM_0.1_ range) induced a strong inflammatory response compared to larger particles [13,14]. Other studies have indicated that PM_10_ with higher endotoxin elicit higher cytokine secretion compared to PM_2.5_ [15], indicating that endotoxin may be a more important determinant. The nature of the inflammatory response may also differ, depending on the particle size. While all sizes (coarse, fine and ultrafine) induced IL-8 release from A549 cells, particles in the fine and ultrafine category induced higher IL-6 compared to coarse particles [12].

Inhalational exposure to PM_0.1_ is commonly associated with enhanced pulmonary inflammation that is characterized by the rapid influx of neutrophils and induction of pro-inflammatory cytokines. PM_0.1_ can also reach the alveolar region, which is the primary site of gas exchange in the lung [16]. It is noteworthy that atherosclerotic plaque formation in mice is enhanced by exposure to ambient PM_0.1_ due in part to the promotion of systemic pro-inflammatory mediators [17], an effect that may be due to direct activation of endothelial cells [18]. PM_0.1_ of particular interest include cerium oxide (CeO_2_), a NP often added to diesel fuel to increase burning efficiency, is now associated with adverse health outcomes. Inhalational exposure of CeO_2_ to CD1 mice significantly increased lung neutrophils and the pro-inflammatory cytokines tumor necrosis factor-α (TNF-α) and interlekin-1β (IL-1β) [5]. Sub-acute inhalation of PM_0.1_ also increases pulmonary inflammation in mice and is thought to be a starting point for long-term cardio-pulmonary health effects [19]. The effects of PM_0.1_ may extend beyond their ability to incite inflammation, and lead to significant impairment of recruited and resident inflammatory cells. For example, exposure of human alveolar macrophages to PM_0.1_ impairs their phagocytic ability, which may increase susceptibility to infections in those with underlying disease (e.g., asthma, COPD) [20].

### 2.2. Oxidative Stress and Antioxidant Defense

Oxidative stress and inflammation are closely related processes that can be induced by each other. Oxidative stress arises from an imbalance between oxidants and antioxidants that ultimately lead to the generation of reactive oxygen (ROS) and reactive nitrogen species (RNS) [21]. ROS are a class of molecules deprived of a complete electron pair and include free radicals and oxidants [21]. Free radicals are generally small molecules with an unpaired electron in the outer valance and include the superoxide anion (O_2_^−^), hydroxyl radical (^•^OH) and hydrogen peroxide. ROS are produced by all aerobic organisms during cellular respiration. In immune cells, such as neutrophils and mast cells, there is a respiratory burst and hence increased release of ROS. Oxidative stress is one of the most common cellular outcomes associated with PM_0.1_ toxicity. Increased ROS due to exposure to PM_0.1_ have been shown in various cell types, including those of the respiratory system such as normal bronchial epithelial cells (NHBE) [22], Beas-2B [23,24,25], A549 [22] and human lung fibroblasts [26,27]; those with relevance to cardiovascular disease include human umbilical vein and human aorta endothelial cells (HUVEC/HAEC) [28,29] and macrophages [30]. Exposure of mouse pulmonary microvascular endothelial cells to an ambient PM_0.1_—with or without cigarette smoke extract (CSE)—induced oxidative stress that activated the mitogen-activated protein kinases (MAPKs) to induce the pro-inflammatory cytokine IL-6 [31]. PM_0.1_ are the most potent at inducing oxidative stress compared to coarse and fine particles; due to their small size, they can enter the cell and damage the mitochondria, leading to ROS production [32].

Because they are highly reactive, ROS can damage key cellular components such as DNA, proteins, mitochondria and lipids. Thus, if cellular repair mechanisms cannot compensate for the excess oxidative stress and consequent cellular damage, then cell death can occur by necrosis (non-physiological) or apoptosis (programmed cell death) [33]. Indeed, exposure to PM_0.1_ increases oxidative stress and DNA damage with no compensatory upregulation of DNA repair in elderly populations [34] and neonatal animals [35], indicating the potential for disease in susceptible populations [36]. The production of ROS are counter-balanced with biochemical antioxidants [37]. A crucial part of the antioxidant system includes glutathione (GSH), a nonprotein tripeptide assembled from cysteine, glutamic acid and glycine via the enzymes glutamate cysteine ligase (GCL) and glutathione synthetase. Thiol groups present on the cysteinyl moiety of GSH react with oxidants and oxidized proteins, thereby detoxifying them. This reaction generates an oxidized dimer of GSH-GSSG that is reduced back to GSH by glutathione reductase. GSH participates in many important antioxidant defense pathways including the detoxification of superoxide (O_2_^−^) via the enzyme superoxide dismutase (SOD). Indeed, there are significant perturbations in GSH levels with exposure to PM_0.1_ [38,39]. Thus, an oxidant-antioxidant imbalance can lead to significant alterations within the cell that can culminate in enhanced cell death mechanisms.

### 2.3. Cell Death Pathways

#### 2.3.1. Necrosis

Among the most commonly reported modes of cell death related to PM exposure are necrosis, apoptosis and necroptosis. Necrosis is characterized by cell swelling and loss of membrane integrity that has recently been classified as regulated necrotic cell death (necroptosis) and accidental cell death (necrosis) [40]. Necroptosis reflects both the programmed nature of this pathway, which shares characteristics with apoptosis (see below) and its necrotic morphological features, namely swelling and lysis of the cell. Necroptosis signaling involves the kinase activity and subsequent physical interaction of receptor interacting protein kinases 1 and 3 (RIPK1/RIPK3) to form the necrosome, which then subsequently mediates the phosphorylation of mixed lineage kinase domain-like proteins (MLKL). Following their activation, p-MLKLs then dimerize, subsequently interacting with, and establishing, ion channels in the cell membrane, thus enhancing permeability that results in cell swelling and lysis. There have been several reports of PM_0.1_-induced necrotic cell death. Foldbjerg and colleagues [41] observed that upon treatment of a human monocytic cell line (THP-1 cells) with polyvinyl pyrrolidone-coated silver NPs (AgNPs), higher doses (>2500 mg/mL) induced necrotic cell death whereas lower doses (<2500 μg/mL) induced apoptosis. Similar to this, Pan and colleagues (2009) observed that the size of the gold NPs determines both their toxicity as well as the cell death mechanism [42]. Here, exposure to smaller AuNPs (1.4 nm in diameter) were significantly more cytotoxic, inducing oxidative stress and ultimately necrotic cell death, whereas larger AuNPs (15 nm in diameter) were deemed non-toxic and associated with apoptotic cell death.

#### 2.3.2. Apoptosis

Apoptosis is a tightly regulated molecular process morphologically associated with cellular fragmentation, membrane blebbing and shrinkage. Apoptosis is necessary during development, cell turnover, and immune system regulation. Dysregulation of apoptosis has been implicated in disease etiology, and in particular, during the emphysematous component of COPD. PM_0.1_ directly affect cell apoptosis. In the human alveolar epithelial cell line A549, treatment with a soluble fraction of ambient PM_0.1_ induced high levels of ROS and consequent apoptosis [43]. Apoptosis can occur through the extrinsic or intrinsic pathways. The extrinsic pathway begins by activation of a “death receptor” by soluble cytokines such as tumor necrosis factor-α (TNF-α) to promote a “pro-death” pathway through c-Jun and the processing of pro-caspase 8 into caspase-8, where it cleaves caspase-3, a key executor of apoptosis. The intrinsic pathway involves the mitochondria, where there is disruption in mitochondrial membrane potential and release of cytochrome C, which activates caspases. The BAX family of proteins sense mitochondrial health, and execute the apoptotic cascade when mitochondrial membrane potential drops. Bcl-2, which gates the mitochondria, is downregulated by Bax allowing cytochrome C to translocate from the mitochondria into the cytoplasmic space. The translocation of cytochrome C is a critical event that triggers the activation of caspase-9, which leads to caspase-3 activation. Thus, activation of caspase-3 results in catalytic processing, and cleaved caspase-3 then coordinates the degradation of proteins involved in cellular assembly and repair including poly (ADP-ribose) polymerase (PARP) and lamin A/C. There is evidence for apoptosis upon exposure to PM_0.1_. Exposure of rat epithelial lung cells to carbon black (in diesel engine emissions and forest fires) induces apoptosis through activation of c-Jun kinase (JNK), a kinase that phosphorylates the apoptotic factor c-Jun [44]. This effect can be seen in vivo, where exposure in mice to ultrafine colloidal silica particles (UFCSs) increases markers of oxidative stress and apoptosis [45].

### 2.4. Autophagy

Autophagy is a form of programmed cell that involves the cytoplasmic uptake and subsequent lysosomal degradation of damaged protein, substrate and organelles. In response to ATP depletion, numerous autophagy-related (Atg) proteins are activated and recruited to the pre-autophagosomal structure (PAS), which initiates the formation of a double membraned autophagic vacuole (AVI) known as the autophagosome. During maturation, the autophagosome and its enveloped cargo undergo a fusion event with a lysosome, rendering the newly synthesized structure an autophagolysosome (AVII) [46]. Dysregulation of autophagy is implicated in many diseases, and there are numerous examples of PM_0.1_ inducing autophagy. Ceria NPs and quantum dot nanocrystals have been considered inducers of autophagy [47,48]. Li and colleagues, for example, illustrated that human lung fibroblasts are able to internalize these AuNPs, which subsequently induce oxidative stress and autophagy [49]. In a recent study, blockage of autophagy reduced the expression of inflammatory cytokines in mouse airways exposed to PM_0.1_. This study also demonstrated that mice with impaired autophagy had significantly reduced airway inflammation and mucus hyper-production in response to the PM_0.1_, suggesting that autophagy contributes to PM-induced airway epithelial injury and inflammation [50].

## 3. Signal Transduction Pathways Implicated in PM_0.1_-Induced Inflammation and Cell Death

### 3.1. Nuclear Factor-κB (NF-κB)

NF-κB is composed of five proteins: p65 (RelA), p50 (NF-κB1), p52 (NF-κB2), c-Rel and RelB [51]. The classic NF-κB pathway is composed of p65/p50 heterodimers, where in response to inflammatory stimuli, including cigarette smoke, this heterodimer translocates to the nucleus to increase inflammatory protein expression [52]. Therefore, the NF-κB pathway is of potential importance in PM_0.1_-induced toxicity. In HUVECs for example, silica NPs induced ROS production and downstream activation of NF-κB, which ultimately caused apoptotic cell death and the release of inflammatory cytokines [53]. Cu-NPs also increased phosphorylation of IκBα and p65 in vivo, indicators of activation, in the liver [54]. In a study in mouse macrophages, Nishanth and colleagues evaluated the inflammatory response to various PM_0.1_ including Ag, aluminum (AI), carbon black, carbon-coated Ag (CAg) and Au, and describe the capability of these PM_0.1_ to increase the expression of IL-6 and COX-2 and induced nuclear translocation of NF-κB [55]. Exposure to ZnO to human bronchial epithelial cells increased IL-8 production, a pro-inflammatory cytokine that promotes the recruitment of neutrophils to target organs, in an NF-κB-dependent manner [56]. The ability of PM_0.1_ to activate NF-κB may be due in large part to their ability to generate ROS production, as pretreatment with the antioxidant and glutathione precursors *N*-acetyl-l cysteine (NAC) blunts NF-κB activation [55]. The effect of the PM_0.1_ on NF-κB activity may also depend on the interaction with other agents. Although AuNP, for example, increases NF-κB activity, when combined with a toll-like receptor 9 (TLR9) ligand, there is suppression of NF-κB activity and subsequent TNF-α production [57]. Thus, in addition to inflammation-enhancing effects, impairment of innate immunity by PM_0.1_ via the NF-κB pathway should also be considered. In this context, pre-exposure of mice to carbon black decreased RSV virus-induced expression of TNF-α, inducible protein (IP-10) and interferon γ (IFN-γ) but increased the expression of the Th2 cytokine interleukin (IL)-13 in the lungs of RSV infected mice, suggesting that pre-exposure to PM_0.1_ induces an inflammatory Th2 environment rather than Th1, thereby promoting an allergic immune response rather than one for microbial defense. 

Activation of the canonical (RelA/p65) pathway also increases RelB expression [58], a REL protein of the alternative NF-κB pathway. The alternative NF-κB pathway is made of p52/RelB proteins. RelB is sequestered in the cytosol by p100 that is processed to p52, which releases p52/RelB heterodimers, which then translocate to the nucleus [59]. The biological functions of the alternative NF-κB pathway are diverse and include thymic and secondary lymphoid organogenesis as well as B cell development [60]. The regulation of the alternative NF-κB pathway can occur via stabilization of NIK, which increases NIK expression to allow efficient processing of p100 to p52 [61]. Inflammatory stimuli can also increase RelB expression, nuclear localization [11,58,62] or cleavage of RelB [63,64], which may be how RelB controls inflammation in the liver and lung [65,66]. RelB potently controls pulmonary inflammation in response to cigarette smoke [67]. By increasing pulmonary RelB via adenoviral delivery, we were able to demonstrate the importance of RelB in reducing neutrophilia in response to cigarette smoke inhalation [67]. At the time of this writing, there were no published reports on the regulation of the non-canonical (RelB) NF-κB pathway by PM_0.1_.

### 3.2. Aryl Hydrocarbon Receptor (AhR)

The AhR is well known to mediate the toxic effects of the man-made environmental contaminant 2,3,7,8-tetrachlorodibenzo-p-dioxin (TCDD; dioxin). When the AhR binds to dioxin, the AhR disassociates from its chaperones (e.g., hepatitis B virus X-associated protein 2 (XAP2), heat shock protein 90 (Hsp90)), translocates to the nucleus and heterodimerizes with the aryl hydrocarbon receptor nuclear translocator (Arnt), followed by binding to DNA sequences termed the dioxin response elements (DRE). This sequence of events leads to the transcription of cytochrome P450 enzymes such as CYP1A1 and CYP1B1 as well as the aryl hydrocarbon receptor repressor (AHRR). The AHRR is a negative regulator of its own pathway by inhibiting AhR function through competing with the AhR for dimerization with Arnt [68,69]. The involvement of the AhR in PM_0.1_ pathology is not yet clear. Although we have shown that the AhR protects against cigarette smoke-induced pulmonary oxidative stress, apoptosis and inflammation [11,70,71,72], and that cigarette smoke contains large amounts of PM_0.1_ [73], we cannot ascribe a protective function regarding protection against PM_0.1_ in smoke. However, premixed flame and environmental particles (PFP 70-nm) cause lung cytotoxicity in neonatal animals, which have less expression of CYP1A1 and CYP1B1, also suggesting a protective role for the AhR against environmental PM_0.1_ exposure [74].

### 3.3. Nuclear Factor (Erythroid-Derived 2)-Like 2 (Nrf2)

Nrf2 is a transcription factor that facilitates the transcription of a battery of genes for antioxidant and phase II metabolic defense including heme oxygenase-1 (HO-1), superoxide dismutase-1 (Sod1), sulfiredoxin-1 (Srxn1) and NAD(P)H:quinone oxidoreductases (Nqo1) [10]. Nrf2 is held in the cytoplasm by its repressor kelch-like associated protein 1 (Keap1), which ubiquitinates Nrf2 for proteosomic degradation. As such, in the absence of stress, Nrf2 has a short half-life (10–30 min) and is expressed at low levels due to continual proteolytic degradation. Upon oxidative insult, however, thiol groups on Keap1 are oxidized, liberating Nrf2 and allowing for its nuclear translocation. After dimerization with MAF, Nrf2 binds to a specific sequence of DNA known as the antioxidant response element (ARE) present in the promoter regions of genes that code for antioxidant and phase II detoxifying enzymes. Dysregulation of Nrf2 expression and function, concomitant with heightened oxidative stress, has been implicated for many chronic diseases including COPD. Metal PM_0.1_, including Ag, Au, NiO, TiO_2_ and CeO_2_, have been investigated for their ability to induce oxidative stress in association with the regulation of Nrf2. In Beas-2B cells, CeO_2_ increases oxidative stress and induced the translocation of Nrf2 to the nucleus and increased HO-1 expression [23]. In studies by the same group, a non-metal oxide PM_0.1_ such as silicon dioxide (silica) also increases ROS production, translocation of Nrf2 and induction of antioxidant proteins [24]. In an animal model of daily intratracheal instillation of TiO_2_ NP, there was significant induction of Nrf2 mRNA and protein in the lung through 75 days; there was also significantly more HO-1 and the catalytic subunit glutamate-cysteine ligase (GCLC) levels, the rate-limiting enzyme involved in the synthesis of GSH [75]. Some of the long-term effects may dependent on Nrf2 expression in specific cell types. Li et al. showed that Nrf2 deficiency in dendritic cells (DCs) provoked a more severe allergic inflammation in the lung of mice after exposure to ovalbumin simultaneously with ambient PM_0.1_, suggesting that Nrf2 deficiency in DCs increases the adjuvant effect of PM_0.1_ on allergic sensitization [76]. Exposure to urban vehicular PM_0.1_ induces the expression of phase II enzymes (e.g., NQO1 and HO-1) and increases the expression and the nuclear translocation of Nrf2 in brain (cerebellum), liver, and lungs only in young (but not older) mice, suggesting that protection against oxidative stress caused by urban PM_0.1_ exposure decreases with age, which may contribute to the development of chronic and systemic disease [77].

### 3.4. PI3K/Akt/mTOR

Phosphoinositide 3-kinase (PI3K) serves as the upstream activator of protein kinase B (PKB, also known as Akt kinase), which together are involved in an intracellular signaling cascade that cumulatively have a pro-survival effect. The mammalian target of rapamycin (mTOR) is an evolutionarily conserved serine-threonine protein kinase and a member of the PI3K family that negatively regulates autophagy. Aberrant PI3K/Akt/mTOR signaling is evidenced in several pathologies, including atherosclerosis. Given the previously discussed role of autophagy in respect to PM_0.1_ exposure, coupled with the ability of the PI3K/Akt/mTOR signaling pathway, a next logical step would be to assume an association between this pathway and PM_0.1_ exposure. Indeed, Roy and colleagues illustrated the mechanism through which ZnO NPs induce macrophage cell death by downregulating PI3K/Akt/mTOR phosphorylation, which functionally leads to enhanced autophagic and apoptotic cell death [78]. Similar findings were illustrated by Duan et al. (2014) in response to endothelial cell exposure to silica NPs, the exposure to which results in an inhibition of PI3K/Akt/mTOR signaling and a subsequent increase in autophagic cell death [79]. Although few studies have examined the effects of ambient PM_0.1_ in vivo, Liu and colleagues (2011) addressed this by assessing the effects of exposure to functionalized single-walled nano-tubes. In vitro, these NPs resulted in changes in PI3K/Akt/mTOR signaling and in vivo resulted in acute lung injury [80]. Taken together, these studies illustrate activation of a common signaling pathway in response to different NPs.

### 3.5. Transcription Factor EB (TFEB)

Transcription factor EB (TFEB) is the master of the regulatory gene network (CLEAR) [81]. This transcription factor, as a global modulator of intracellular clearance and energy metabolism, lysosomal biogenesis, has provided new insight into the mechanism by which the cell responds to environmental stimuli [48,82]. TFEB is a master regulator of lysosomal biogenesis, autophagy and cell adaptation that regulates the expression of genes encoding lysosomal proteins [83,84], the processing of lysosomal proteins and the expression of autophagy genes [82,85]. TFEB was proposed to be a coordinator of autophagy and lysosomal functions in cells exposed to PM [47]. Here, ceria NPs functionalized with different types of biocompatible coatings (e.g., *N*-acetylglucosamine, polyethylene glycol and polyvinylpyrrolidone) activated TFEB-regulated genes of the lysosome-autophagy system. These investigators also showed that the array of differently functionalized ceria NPs enhanced autophagic clearance of proteolipid aggregates that accumulate as a result of inefficient function of the lysosome-autophagy system. Translocation of TFEB from the cytosol into the nucleus is a prerequisite for gene regulation and represents an example that this cellular process is induced by PM. There is currently no information on TFEB regulation by PM from other sources such as air pollution or cigarette smoke.

## 4. Epigenetic Contribution to Pulmonary and Systemic Disease

**Figure 2:**
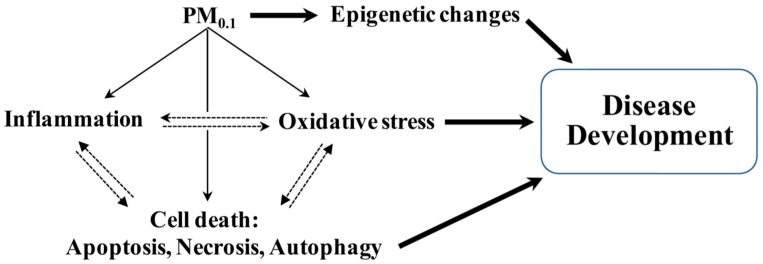
Intersecting pathogenic mechanisms converge to increase susceptibility to developing chronic cardiopumonary diseases assocoated with exposure to PM_0.1_.

Toxicogenomics has emerged as a field that combines traditional toxicology with functional genomics to define adverse effects of environmental contaminants. Such characterization involves quantification of changes at the DNA and RNA level but more recently involves analysis of microRNA (miRNA) expression and other epigenetic changes including DNA methylation, histone modifications, and other non-coding RNAs. Epigenetics refers to heritable changes in gene expression without accompanying alterations in the DNA sequence [86]. Such epigenetic alterations can include DNA methylation, histone tail modification as well as non-coding RNA (ncRNA)-mediated events [87] and may underlie the pathogenesis of complex diseases associated with environmental exposure to PM_0.1_ (Figure 2). 

### 4.1. MicroRNA (miRNA)

miRNAs constitute a large group of non-coding RNAs that downregulate protein expression by triggering translational repression and/or mRNA degradation [88]. miRNAs are synthesized as longer transcripts which are then processed by the ribonucleases Drosha and Dicer, giving rise to mature miRNAs. Mature miRNAs assemble with members of the argonaute protein family into miRNA-induced silencing complex. The miRNA directs the RNA-induced silencing complex (RISC) to target mRNAs for degradation [89]. Aberrant expression of miRNA is now implicated in many diseases, and there is increasing interest in the use of miRNA signatures as biomarkers for diseases such as cancer and COPD [90,91,92,93]. The most studied environmental factor for miRNA is smoking. Changes in miRNA levels in response to cigarette smoke exposure—the main cause of COPD for example—is well-noted in vitro as well as in vivo after smoke exposure and in individuals with COPD [90,94,95,96]. Changes in miRNA levels after exposure to diesel exhaust particles (DEP) are also evident in human lung cells [97]. As both cigarette smoke and diesel exhaust contains large amounts of PM_0.1_ [73,98], it remains to be seen whether these changes in miRNA are related to particle composition or size, especially in light of the chemical nature of complex mixtures (e.g., cigarette smoke, DEP). Thus, it is likely that PM_0.1_ contribute only a component towards the regulation of miRNA expression.

However, the effects of individual PM_0.1_ can also mediate changes in miRNA expression in experimental systems and may even be useful for detecting exposure to PM_0.1_ as potential biomarkers. Using AuNPs administered intravenously to adult Wistar rats, Chew and colleagues reported on the differential regulation of 23 blood miRNA levels, with signatures being both similar and different based on 1 week versus 2 months post-exposure [99]. It was noteworthy that some miRNA remained significantly changed at both time points including miR-146b, which was significantly decreased with exposure to AuNP. Members of the miR-146 family (miR-146a and miR-146b) are negative regulators of inflammation [94,100,101] and are typically increased as a means to attenuate inflammatory protein expression (e.g., IL-8, COX-2). Thus it is interesting to speculate the NP-induced changes in anti-inflammatory miRNA such as those belonging to the miR-146 family, may significantly contribute to alterations in inflammation. Additional studies using TiO_2_ inhalation in mice has revealed significant changes in lung inflammatory cells (predominantly increased neutrophilia), cytokines and acute phase proteins [102]. This same study also performed miRNA expression profiling and reported that 55 miRNA in the lungs were also altered as a consequence of TiO_2_ exposure, including those associated with immunological responses such as miR-21 [102]. TiO_2_ is not the only PM_0.1_ that can cause changes in miR-21. Carbon black NP also induced pulmonary expression of miR-21, as well as miR-135b and miR-146b, all of which were increased following exposure [103]. However, pulmonary administration of carbon black did not significantly alter cardiac miRNA expression. Moreover, incubation of mouse epidermal cells with Tungsten carbide-cobalt (WC-Co) NPs increased miR-21 [104]. It is intriguing that in the former study, miR-146b in the lung is increased, whereas it was decreased in the blood. Whether this difference in miR-146b levels is reflective of the PM_0.1_ used (AuNP versus carbon black), the species (rat versus mouse) or the target organ (blood versus lung) remains to be seen. While bioinformatic analysis in search of predicted miRNA target proteins (e.g., using TargetScan (http://www.targetscan.org/)) indicates potential targets of these miRNA, there remain exceedingly few studies to directly evaluate whether the PM_0.1_-induced changes in select miRNA directly regulate protein expression and hence contribute to pathogenic processes described above. Despite little evidence in the literature validating PM_0.1_-induced miRNA changes, miRNA expression profiles are offering potential as biomarkers of organ-specific damage. A recent example of this includes analysis of organ-specific miRNA such as miR-122, miR-192 and miR-194; miRNA that are highly expressed in the liver may also serve as marker of organ damage by silica NP. Administration of silica NP at a diameter of 70 nm induced severe liver damage in mice and significantly increased systemic miR-122 [105,106]. Thus, accumulating evidence supports that that environmental agents, including PM_0.1_, contribute significantly to alterations in miRNA levels.

### 4.2. Long Non-Coding RNA (lncRNA)

Long non-coding RNAs (lncRNAs) constitute a large of family of RNAs greater than 200 nucleotides long [107]. Originally, it was believed that lncRNA constituted “transcriptional noise,” and thus only synthesized incidentally with the transcription of traditional protein-coding genes. Now it is known that lncRNAs possess a diverse repertoire of cellular functions, some of which may play crucial roles in the pathophysiology of air pollution-induced disease. First, lncRNAs can influence the transcription of protein-coding genes via antagonization of RNA polymerase II and the transcription initiation complex [108]. Second, lncRNAs regulate gene expression at the post-transcriptional level by masking splice sites on mRNAs [109], preventing miRNA binding, and even sequestering large numbers of miRNA species by functioning as “miRNA sponges” [110]. Finally, lncRNAs can influence the function of translated proteins via sequestering these proteins [111] and/or providing scaffolding for multi-protein complexes [112]. However, there remains limited information on the role lncRNAs play in response to particulate matter, including PM_0.1_. One study by Thai and colleagues revealed the lncRNA SCAL1 is induced in vitro in lung cancer cell lines by cigarette smoke extract (CSE). Further analysis of the mechanism revealed that CSE induced Nrf2-dependent binding to the promoter of SCAL1 for transcriptional upregulation and that SCAL1 was required for cell survival [113]. The contribution of this lncRNA—or that of others—in the pathogenesis of diseases associated with PM_0.1_ is unknown.

### 4.3. DNA Methylation

DNA methylation is one of the most-studied epigenetics events that can influence cell behavior by promoting or inhibiting transcription. It is also heritable by somatic cells after cell division, and is essential for embryogenesis, with methylation pattern changing to accommodate differentiation and development [114]. There are numerous studies that have examined epigenetic effects, specifically DNA methylation, in response to fine and ultrafine air pollution particles (reviewed in [87]). In workers exposed to PM_10_, for example, there was lower promoter methylation of inducible nitric oxide synthase (iNOS) [115]. Cigarette smoke has been widely studied as a model of epigenetic changes, where DNA hypermethylation of CpG islands in the promoter region of tumor suppressors and other genes has been described [116]. Moreover, long-term exposure to aerosol PM was negatively associated with global methylation of the DNA [115]. It is noteworthy that lower methylation of DNA is found in patients with cancer or cardiovascular disease [117], suggesting that lower DNA methylation is a possible mechanism by which exposure to PM_0.1_ can lead to the development of disease.

One of the pathways affected by environmentally-induced methylation involves the AhR and specifically, the AHRR. In addition to being a negative regulator of AhR activity (reviewed above), it has been suggested that the AHRR is a tumor suppressor and is downregulated in many types of cancer due to DNA hypermethylation [118]. The association between AHRR methylation/smoking remains the most convincing example of a relationship between environmental exposures and DNA methylation changes in humans [119]. In this light, there are numerous studies that have shown an association between smoking and AHRR methylation status [120], such that methylation changes occur early [121]. Furthermore, methylation levels are close in former smokers when compared to never smokers [122], suggesting some reversibility in the process. Decreased methylation of the AHRR in smokers is associated with decreased expression in lymphoblasts [120], is affected by maternal smoking [119], and may represent a biomarker of smoke exposure [121]. While at the time of this writing there is no information on the methylation status of the AHRR in response to other forms/sizes of PM (e.g., PM_0.1_), it is possible that AHRR methylation may be a general response rather than specific to cigarette smoke. In support of this, there is emerging evidence that several types of engineered PM_0.1_ can induce global DNA hypomethylation, including SiO_2_ in HaCaT cells, [123], and that hypermethylation of the poly-ADP-ribose polymerase-1 (PARP-1) promoter corresponds with decreased PARP-1 gene expression [124].

These long-term environmental exposures may also significantly impact the health of subsequent generations. Studies now indicate that maternal exposure to cigarette smoke, for example, impacts molecular pathways in newborns due to methylation of CpG sites, including the AhR pathway [125,126]. This epigenetic signature also persists into childhood when exposed in utero [127]. Such changes may impact disease susceptibility later in life. Studies using experimental models of asthma indicate that in utero exposure to smoke caused significant alterations in DNA methylation that was associated with features of asthma (airway hyperactivity, inflammation) [128]. With respect to air pollution, exposure to PM_2.5_ is associated with lower placental DNA methylation [129]. Furthermore, maternal exposure to combustion-derived PM_0.1_ enhances post-natal asthma development in mice by mechanisms speculated to be epigenetic in nature [130]. Additional studies to evaluate potential epigenetic changes associated with PM_0.1_ exposure are warranted.

## 5. Conclusions

Personal exposure to PM_0.1_ (ultrafine particles smaller than 100 nm) occurs on a daily basis, and such chronic exposure is now associated with adverse health effects. Because of their extremely small size, PM_0.1_ are deposited deep into the lungs and subsequently distributed throughout the organism via the circulatory system, reaching secondary organs, such as the brain, heart and liver. At the cellular level, exposure to PM_0.1_ activates several signaling pathways (i.e., NF-κB, NADPH oxidase) to orchestrate pathological responses characterized by production of inflammatory mediators and reactive oxygen species (ROS). This is followed by a cascade of events that lead to the death of cells by different mechanisms (apoptosis, necrosis, autophagy). Particle accumulation due to long-term exposure is thus associated with chronic inflammatory diseases such as COPD. PM_0.1_ also may cause DNA mutations and epigenetic modifications, which can lead the affected cell to transform into cancer cells. Even though research on PM_0.1_ is almost 30 years old, further studies are needed to better understand the mechanisms of toxicity of inhaled urban PM_0.1_ to develop new preventative and/or therapeutic strategies to reduce the negative impact of PM_0.1_ on human health. Future studies of PM_0.1_ should be integrated with physical and chemical research to perform tailored experimental conditions, in addition to addressing key unknown mechanistic processes from air pollutant transformation before and after exposure, alteration on biological surfaces, physiological impacts and their ultimate impacts.
